# Cut&tag: a powerful epigenetic tool for chromatin profiling

**DOI:** 10.1080/15592294.2023.2293411

**Published:** 2023-12-17

**Authors:** Zhijun Fu, Sanjie Jiang, Yiwen Sun, Shanqiao Zheng, Liang Zong, Peipei Li

**Affiliations:** a BGI Tech Solutions Co, Ltd. BGI-Shenzhen, Shenzhen, China; b BGI Tech Solutions Co, Ltd. BGI-Wuhan, Wuhan, China

**Keywords:** CUT&Tag, chromatin profiling, epigenetics, Tn5 tagmentation, single-cell CUT&Tag

## Abstract

Analysis of transcription factors and chromatin modifications at the genome-wide level provides insights into gene regulatory processes, such as transcription, cell differentiation and cellular response. Chromatin immunoprecipitation is the most popular and powerful approach for mapping chromatin, and other enzyme-tethering techniques have recently become available for living cells. Among these, Cleavage Under Targets and Tagmentation (CUT&Tag) is a relatively novel chromatin profiling method that has rapidly gained popularity in the field of epigenetics since 2019. It has also been widely adapted to map chromatin modifications and TFs in different species, illustrating the association of these chromatin epitopes with various physiological and pathological processes. Scalable single-cell CUT&Tag can be combined with distinct platforms to distinguish cellular identity, epigenetic features and even spatial chromatin profiling. In addition, CUT&Tag has been developed as a strategy for joint profiling of the epigenome, transcriptome or proteome on the same sample. In this review, we will mainly consolidate the applications of CUT&Tag and its derivatives on different platforms, give a detailed explanation of the pros and cons of this technique as well as the potential development trends and applications in the future.

## Introduction

Since the first draft of the human genome sequence was released in 2001 [1], an increasing number of researchers have been engaging in genome research. In the postgenomic era, attention has been given to mapping the protein components and chromatin modifications into the human genome as well as the genomes of other eukaryotes. An analysis of transcription factors and chromatin modifications at the genome-wide level provides insights into gene regulatory processes, such as transcription, cell differentiation and cellular response. Transcription factors regulate the transcriptional levels of virtually all genes by targeting specific genomic loci and reading the DNA code. Transcription factors are divided into two types: DNA-binding transcription factors and non-DNA binding cofactors [2]. DNA-binding transcription factors directly recognize and bind to specific DNA motifs to mediate gene transcription, while transcriptional cofactors interact with DNA-binding transcription factors, which involves the stabilization of transcription factors on DNA, the modification of chromatin structure and the positive or negative regulation of gene transcription.

However, a lack of adequate methodologies for mapping chromatin fragments has prevented epigenomic profiling from realizing its full potential. Since chromatin immunoprecipitation (ChIP) was developed in the mid-1980s, it has become the most popular and powerful approach for chromatin analysis [3,4]. During ChIP, chromatin is cross-linked and sonicated into small fragments in solution. Next, an antibody is used to pull down chromatin epitopes. Genomic DNA is then extracted and subjected to high-throughput deep sequencing. Due to cross-linking and sonication, ChIP-seq and its variations are characterized by a large number of cell inputs, epitope masking, high background and low signal-to-noise. In addition to ChIP, other enzyme-tethering techniques have recently become available for living cells, including pA-DamID [5], ChEC-seq [6], CUT&RUN [7], the single-molecule method BIND&MODIFY [8] and Cleavage Under Targets and Tagmentation (CUT&Tag) [9], which target the chromatin of a specific protein of interest in situ and then profile its genomic distribution. The CUT&RUN protocol uses the fusion of MNase and protein A (pA-MNase) to cleave chromatin across the genome, which becomes a binding site for specific antibodies. Compared with ChIP-seq, CUT&RUN requires as few as 100–1000 cells, greatly reduces genome-wide profiling costs and provides base-pair resolution of chromatin constituents with a much lower background. Nevertheless, DNA end repair and adapter ligation are necessary for sequencing library preparation, which results in a longer time-consuming, more costly, and more labour-intensive process. Using recombinant protein A-M.EcoGII methyltransferase, BIND&MODIFY labels and methylates the adenines of local DNA regions in a m6A non-specific manner without fragmentation, then nanopore is performed to detect the protein-binding motif with artificial m6A modifications. It is possible to measure CpG 5mC methylation, histone modification status, and transcription factor binding at the same time [8]. However, several drawbacks of this novel technology should be considered, including the base calling accuracy of nanopore sequencing and endogenous methylation.

With the success of CUT&RUN, CUT&Tag was further improved by using a protein A/G fused to a Tn5 transposase preloaded with DNA sequencing adapters instead of the pA/G-MNase in 2019 [10]. In the CUT&Tag experiment, the primary antibody recognizes the target protein and then mediates the binding of the secondary antibody and the protein A/G-Tn5 fusion protein. Tn5 can precisely target and cleave the DNA sequence near the target protein in the presence of Mg^2+^. Simultaneously, the sequencing adapters will be inserted into both ends of DNA fragments by Tn5. After PCR amplification, the DNA library can be directly sequenced in a day. Tagmentation is a process that differs from ChEC and CUT&RUN in that it does not require precise control of MNase time and temperature. Unlike the former methods, CUT&Tag integrations are completed at 37°C without any further adjustments. Similar to other chromatin profiling methods, CUT&Tag has some advantages over ChIP-seq: it requires low inputs as few as 100 cells with lower background, higher signal-to-noise, greater repeatability and is a shorter process [11]. CUT&Tag has been applied to the profiling of transcription factors and chromatin modifications in distinct species, including humans [12], mice [13], piglets [14], bovine [15,16], zebrafish [17], Drosophila [18], *Toxoplasma gondii* [19], and plants [20,21].

In this review, we will mainly consolidate the applications of CUT&Tag and its derivatives on different platforms, give a detailed explanation of the pros and cons of this technique as well as the potential development trends and applications in the future.

## CUT&Tag profiling of histone modifications

Histone posttranslational modifications refer to the functional set of epigenetic marks that act as direct or indirect effectors to contribute to activating the cell signalling pathway [22], impeding the access of remodelling complexes, or mediating the recruitment of TFs and chromatin modifiers [23,24]. These modifications consist of methylation, acetylation, phosphorylation, lactylation, ADP ribosylation, ubiquitinylation, sumoylation, deamination, citrullination, butyrylation and propionylation [25]. Genome-wide analysis of histone modifications is essential for shedding light on cellular processes. Alterations in the patterns of histone modifications have been identified at the global level of the genome by using ChIP-chip, ChIP-seq, CUT&RUN and CUT&Tag [26,27].

CUT&Tag was first introduced by Kaya-okur et al. in 2019. An antibody against lysine-27-trimethylation of the histone H3 tail (H3K27me3), a marker of silenced chromatin regions was incubated with human K562 cells [10]. To reduce DNA accessible site in tagmentation by pA-Tn5, CUT&Tag contained 300 mM NaCl during incubation, tagmentation and washes, and then the DNA library was subjected to multiplex paired-end sequencing. The analysis of approximately 8 M reads mapped to the human genome assembly suggested a clear pattern of chromatin sites marked by H3K27me3. Compared with H3K27me3 profiling by CUT&RUN and by ChIP-seq, CUT&Tag had similar landscapes as these two techniques, and the background noise of CUT&Tag and CUT&RUN was significantly lower than that of ChIP-seq. In addition, they obtained genomic profiling of H3K4me1 and H3K4me2, which represent active chromatin regions. The results revealed that CUT&Tag profiling offered stronger signal accumulation at the top 10,000 peaks defined by MACS2 and a lower background than ChIP-seq.

Uveal melanoma(UM) is the most common and life-threatening ocular malignancy. Using CUT&Tag assay, Gu et al. found that disruptor of telomeric silencing-1-like (DOT1L), a methyltransferase of histone H3 lysine 79 (H3K79) contributed to H3K79 methylation of nicotinate phosphoribosyltransferase (NAPRT) and induced NAPRT activation, thereby promoting the malignance of UM [28]. Sumimoto et al. conducted CUT&Tag with histone H3K27ac antibody to reveal that EGFRHDAC mediated the silencing of chemokine genes through chromatin conformational changes, which were associated with immune checkpoint inhibitors (ICIs) resistance by remoulding the T-cell deserted tumour microenvironment in human lung adenocarcinoma [29]. Lactylation of lysine residues of histones (Kla) was first discovered in 2019 [30]. It has been reported that lactylation of histone 3 on lysine residue 18 (H3K18la) highly accumulates on gene promoters and involves gene activation of the related genes in macrophages and cancer cells [31,32]. Galle et al. investigated the genome‑wide distribution of H3K18la by using CUT&Tag in both *in vitro* and *in vivo* samples. The results showed that global H3K18la profiling was similar to H3K27ac profiling, which was distributed at active enhancers that are functionally critical for the respective tissue [33].

## CUT&Tag profiling of transcription factors

TFs play a role in various biological processes. Multiple knockout studies over the last decades have demonstrated that disrupting the function of TFs can completely induce the alterations in gene expression, abnormal tissue formation and change in cell fate [34,35]. In modern biology, one of the most important questions is how TFs shape the epigenetic landscape during cellular development and differentiation. A large number of TFs have not yet been characterized, and the CUT&Tag method has been widely used for TF profiling in research. Compared to histone modifications, slight cross-linking may need to be considered on CUT&Tag profiling of transcription factors, especially for transcription factors that bind weakly to DNA.

Kaya-okur et al. not only implemented chromatin profiling of histone modification by CUT&Tag for the first time, but also applied this method for mapping transcription factor binding. They used an antibody against NPAT nuclear factor, a coactivator of replication-dependent histone genes, to determine whether pA-Tn5 tethered at TFs was differentiated from DNA accessibility sites [10]. Because 80 DNA accessible sites of histone genes on chromosome 1 and chromosome 6 are bound by NPAT [36], they compared these binding sites with DNA accessible sites, about 99% of reads were located at the promoters of histone genes. In order to test whether the CUT&Tag can be utilized to identify more abundant sites of the TF, they determined the profiling of the CCCTC-binding protein by CUT&Tag. They varied the wash buffer stringency to determine its displacement from the chromatin. Undoubtedly, they were able to observe read counts at the various CTCF binding sites, which were highly correlated with the peaks identified by ChIP-seq and CUT&RUN.

β-cell dysfunction and insulin resistance are two major elements to be uncovered in type 2 diabetes (T2D) [37]. Using CUT&Tag assay, Qiao et al. confirmed that Pax6 was an important transcription factor involved in defective GSIS in STING-βKO mice, which suggested the role of STING in regulating insulin secretion and maintaining glucose homoeostasis [38]. Zinc finger protein 143 (ZNF143) is a transcriptional activator that mediates hepatocellular cancer cell cycle transition and cell proliferation [39]. Ye et al. performed CUT&Tag assay with ZNF143 antibody to conclude that the expression level of ZNF143 was critical for the maintenance of cell identity in the liver [40]. The chromosomal translocations of PAX3-FOXO1 or PAX7-FOXO1 TFs are common in an alveolar subclass of rhabdomyosarcoma. Manceau et al. constructed inducible human fibroblast cell lines expressing PAX3-FOXO1 or PAX7-FOXO1 TFs and then performed CUT&Tag for genome-wide profiling of TFs. The results revealed that PAX3-FOXO1 and PAX7-FOXO1 were associated with distinct pathological manifestations in patients [41].

CUT&Tag analysis of many TFs is applied in different biological and pathological processes including odontoblast terminal differentiation [42], embryonic stem cell differentiation [43], blood – testis barrier [44], bladder development [45], Parkinson’s disease [46], acute myeloid leukaemia [47], breast cancer [48,49], lung cancer [50], bladder cancer [51], neuroendocrine carcinoma [52], and mantle cell lymphoma [53].

## Single-cell CUT&Tag

CUT&Tag profiling has demonstrated that the initial cellular material reaches as low as ~ 60–100 cells [10,11]. The technique is further improved to be applied to single cells, providing a unique opportunity to detect epigenome variability between seemingly uniform cell types or within tissues. Unsurprisingly, all 2019 publications describing CUT&Tag were adapted to single cells [10,54]. Although ChIP-seq and CUT&RUN have been reported to perform single-cell chromatin analysis [55,56], due to chromatin fragments in solution, their application is relatively limited. On the contrary, pA-Tn5 of CUT&Tag tightly binds to the DNA at both ends of the antibody targeting fragment, retaining the fragments within the nucleus, so all steps from nuclei or intact cells to fragmentation can be conducted in bulk.

**Figure 1.**
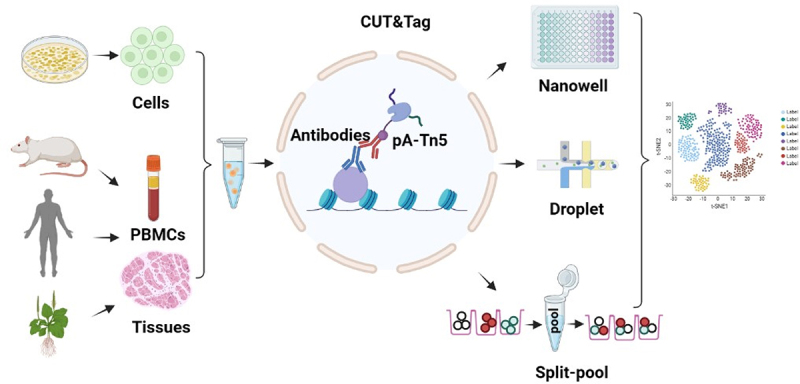
Schematic of scCuT&Tag applied to cultured cells and tissues in different species. Cells or nuclei suspension are isolated from cell culture, mice tissues, human tissues and plant tissues. These cells or nuclei are subjected to antibody incubation, pA-Tn5 binding and tagmentation, then they are combined with nanowell, 10× Genomics or split-pool system to distinguish cellular identity.

The single-cell read-out strategies applied to RNA-seq can also adapt to ATAC-seq and CUT&Tag, where CUT&Tag based on droplets and nanowells utilizes the same barcode strategy, implementation, and analysis software previously developed for ATAC-seq [57,58]. Split-pool approach, which arranges and barcodes the cell population in a 96 well plate, pools and then rearranges them in the new plate, adding a second barcode, and so on [59,60]. This strategy increases the number of cells in one experiment and facilitates multiple experiments to be conducted to relatively reduce batch effects (Figure 1).

scCUT&Tag-based nanowells were developed by Kaya-okur. et al in 2019. H3K27me3 modification was subjected to scCUT&Tag on bulk K562 cells, replacing Concanavalin A magnetic beads with mild centrifugation between steps. After integration, a Takara ICELL8 nanodispensing system was used to divide single cells equally into the nanowells of the 5184-well chip and ensure that the nanowell contained only one cell by imaging the chip. Then, two indexed primers were used to perform PCR assays on each library of each nanowell, and finally, all DNA libraries in the chip were pooled for deep sequencing to evaluate sampling and coverage in each cell with high redundancy. Most of the reads from single cells fallen within the H3K27me3 block defined in bulk analysis, suggesting the high recovery of scCUT&Tag [10]. In another study, the CUT&Tag approach was combined with droplet-based single-cell library preparation to generate high-quality data on single-cell histone modifications. They applied single scCUT&Tag to tens of thousands of cells in the central nervous system of mice to detect the histone modification characteristics of enhancers, promoters, and active genomes (H3K4me3, H3K27Ac and H3K36me3) and inactive regions (H3K27me3). These scCUT&Tag results determined the identity of cells and allowed us to interpret regulatory principles, including promoter duality, H3K4me3 diffusion, and promoter-enhancer connectivity. The single-cell chromatin occupancy of the cohesin complex component RAD21 and TF OLIG2 was also studied by using the scCUT&Tag method [61].

Epigenome analysis is limited to a maximum of two copies of chromatin characteristics per cell. Thus, the strategy for a single-cell epigenome should increase efficiency to get more cell type discriminatory information from each cell. Targeted insertion promoter sequencing (TIP-seq) uses the fusion of Tn5-proteinA incubated with the adaptor of a T7 RNA polymerase promoter inserted upstream of the barcode. Linear amplification of DNA with T7 polymerase and reverse transcription of cDNA prior to sequencing library preparation can result in approximately 10-fold higher unique reads of each cell compared to other methods. TIP-seq has been applied to profiling RNA polymerase II, various histone modifications and the transcription factor CTCF in individual mouse and human cells [62].

## The combinatorial strategy for CUT&Tag and other omics

Due to abundant transcripts expressed from moderately and highly expressed genes, single-cell RNA-seq has rapidly developed. Recent achievements in single-cell technologies have offered enormous promise for multidimensional chromatin analyses, such as scATAC for chromatin accessibility and scCUT&RUN and scCUT&Tag for histone modifications and chromatin binding proteins. Each method only gives a layer of cellular information. To establish connections among these layers, unpaired single-cell omics datasets are integrated by a computational platform [63,64]. However, these strategies acquire prior knowledge-based correspondence between multimodal datasets from different experiments, which limits the ability to reconstruct functional relationships of different informative layers. Therefore, a multimodal single-cell approach that can jointly detect both gene expression and epigenetic features is highly needed.

Xiong et al. developed CoTECH, a combined barcode method that allows high-throughput single cells to jointly identify chromatin sites and the transcriptome [65,66]. In CoTECH, the antibody binding cells is distributed in 96 well plates containing unique combinations of T5 and T7 barcodes assembled by Tn5-protein A fusion protein (PAT). Then, mRNA is reverse transcribed by using oligonucleotide dT primers with specific RNA barcodes. The cells are pooled and redistributed into 96-/384-well plates for preamplification. Samples are purified and divided into two halves for DNA and RNA library preparation, respectively.

Another approach, Paired-Tag, also combines scCUT&Tag and scRNA-seq in the same cell [67]. In this method, cells are subjected to antibody incubation, pA-Tn5 binding and tagmentation. Then, reverse transcription (RT) is performed by using a CUT&Tag well-specific adapter and a well-specific barcode for the RT primer. Finally, cells is pooled and subjected to two rounds of ligation-based split pooling for library preparation in a 96-well plate. Using this method, Zhu et al. performed a combinatorial analysis of five histone modifications and transcriptomes in the frontal cortex and hippocampus of adult mice. Zhang et al. described scCUT&Tag-pro, which enabled profiling chromatin features and the abundance of surface proteins in single cells [68]. Another study introduced CUT&Tag-BS by combining CUT&Tag with bisulphite sequencing to detect joint profiling of histone modifications and DNA methylation in the same sample [69].

## Multifactorial CUT&Tag

CUT&Tag enables genome-wide mapping of the distribution of histone modifications and TFs on chromatin from as few as one cell. The technique only identifies the genomic localization of one chromatin protein at a time, and it is unable to directly measure the colocalization of different chromatin epitopes in the same cells and needs to prioritize targets in the case of limited samples. The combinatorial detection of multiple histone modifications and other chromatin proteins can be achieved by multifactorial CUT&Tag methods.

**Figure 2.**
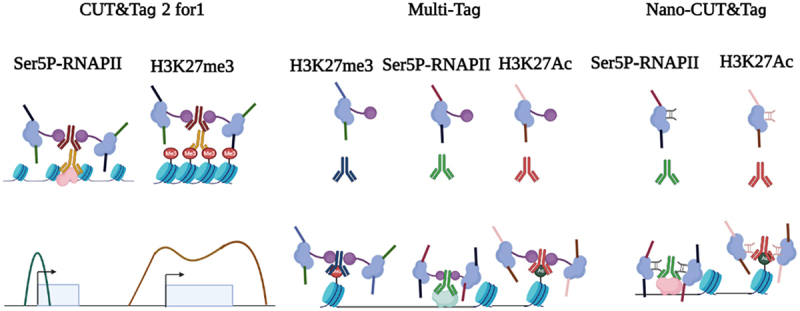
Multifactorial CUT&Tag strategies. (left) CUT&Tag 2 for 1 involves the use of two antibodies against H3K27me3 (heterochromatin marks) and Ser5P-RNAPII (active chromatin marks). This approach follows the same workflow as regular CUT&Tag, except for mixing two antibodies into the cells. The difference in fragment size and feature breadth (broad domains for H3K27me3 and narrow peak of Ser5P-RNAPII) are used together to calculate the deconvolution of two signals. (middle) in MuTi-Tag approach, each antibody is pre-incubated with the barcoded pA-Tn5, thus marking several different histone modifications and TFs in the same experiment. (right) nano-CUT&Tag is the use of nanobodies, single-chain antibodies directly fused to Tn5, where higher efficiency can be obtained without protein A.

CUT&Tag 2 for 1 was designed by using two antibodies against H3K27me3 and initiating RNA polymerase II (Ser5P-RNAPII). H3K27me3 targets silenced Polycomb domains, whereas Ser5P-RNAPII marks promoters and enhancers. Since the heterochromatin regions labelled by H3K27me3 usually span more than 10 kb and are much more condensed in nucleosome spacing, the tagmentation of CUT&Tag will produce larger fragments. In contrast, small fragments (less than 120 bp) will be generated in the active region. This approach follows the same workflow as regular CUT&Tag, except for mixing two antibodies into the permeabilized cells. The difference in fragment size and feature breadth (broad domains for H3K27me3 and narrow peak of RNAPIIS5p) are used together to calculate the deconvolution of two signals [70] (Figure 2).

Further improvements, multi-CUT&Tag and MulTI-Tag, are to incubate barcoded primary antibodies against different chromatin modifications in the same sample. In these methods, each antibody interacts with the barcoded pA-Tn5, thus marking several different histone modifications in the same experiment. Therefore, the location and abundance of each chromatin epitope (such as H3K4me1/2, H3K27me3, H3K27Ac, H3K36me3 and RNAPII) can be recovered by using antibody-specific barcodes, so that several chromatin markers and binding proteins can be simultaneously detected in the same sample. These methods have been successfully applied to single cells to recognize the identity of cell types in cell mixtures [71,72] (Figure 2).

Another important recent development in multifactorial CUT&Tag is the use of nanobodies, single-chain antibodies directly fused to Tn5, where higher efficiency can be obtained without protein A [73,74] (Figure 2). Multifactorial single-cell CUT&Tag is performed by the addition of two nanobody-Tn5 fusions with different barcodes, one specific for mouse primary antibodies and the other specific for rabbit primary antibodies.

## Spatial-CUT&Tag

One of the main obstacles to epigenome technologies (including single-cell method) is the lack of spatial information. Defining the location of chromatin decorated by TFs or specific markers in complex tissues or tumour samples is helpful to illustrate the developmental and biological processes. A recent study reported single-cell Spatial-CUT&Tag. In this approach, a thin tissue section is fixed on a glass microscope slide. After successive incubation with the primary antibody, secondary antibody and pA-Tn5, a 20 μm resolution grid of DNA barcodes is recorded on the slice through microchannel-guided delivery at the cellular and subcellular scales [75].

In the same year, Lu et al. developed another technique named ‘dubbed epigenomic MERFISH.’ Tn5 transposons were first loaded with a T7 promoter in addition to sequencing adaptors. After the antibodies H3K4me3, H3K27me3 and H3K27Ac mediated Tn5 tagmentation and linear amplification, the gene loci could be observed by MERFISH. This method coupled epigenome markers with imaging, powerfully providing the possibility of visualizing the spatial and intranuclear distribution of more than 100 genome sites on tissue slides, including enhancers, promoters, and repressed sites [76]. A strategy for joint profiling of the epigenome and transcriptome on the same tissue section at near-single-cell resolution was reported recently. It is feasible to investigate tissue organization and composition at an unprecedented level of refinement [77].

## Nonprotein-based chromatin profiling by CUT&Tag

CUT&Tag has also adapted to nonprotein characteristics of the genome landscape, such as DNA G-quadruplexes (G4s) and RNA loops. G4s are quadruple helix structures formed by the self-association of guanines. G4s, first identified in 1988 [78], have been reported to be involved in multiple physiological processes, including DNA replication, gene transcription, and epigenetic modifications, telomere elongation and maintenance, providing a new perspective for elucidating the mechanisms of genome stability and gene expression [79–81]. In addition, G4s, as promising drug targets, are associated with neurological disorders, Alzheimer’s disease and cancer [82–84]. R-loops are three-stranded nucleic acid structures formed by RNA-DNA hybrids and displaced single-stranded DNA (ssDNA). Different from RNA-DNA hybrids of transcription, R-loops can span 100–2000 bp behind elongating RNA polymerases. Accumulating evidence reveals that they are negatively associated with DNA replication, genomic stability and transcription, contributing to chromosome segregation and chromatin organization [85–87]. R-loops represent a source of DNA damage, and cells have developed different mechanisms to either prevent or remove hybrids directly by DNA repair [88].

ChIP-seq has been adapted to accurately map the G-quadruplex landscape in the genome by using either a G4-specific antibody BG4 or an artificial 6.7 kDa G-quadruplex binding protein G4 probe (G4P) protein [89–91]. The antibody BG4 is screened by phage-display, while G4P is derived from the protein domain of the DHX36 helicase with specificity and high affinity for G4. Because formaldehyde fixation may result in epitope-masking, Li et al. developed G4 CUT&Tag to profile the genome-wide G4 distribution, offering G4 genome landscape with high resolution, superior signal-to-noise ratio and high specificity in various human cells. The results showed that G4 signatures were cell type-specific and were associated with active epigenetic modifications in different cell lines [80]. Another study used the CUT&Tag assay to profile G4 formation in mouse embryonic stem cells, revealing that widespread G4s were enriched in active promoters as well as poised and active enhancers [92]. Unquestionably, CUT&Tag not only illustrates G4 formations in cell populations but also observes whether G4 features at a specific genomic site are variable between individual cells with single cell G4-CUT&Tag, reflecting that it is feasible to distinguish cellular identity [93].

DNA-RNA immunoprecipitation sequencing (DRIP-seq) has been established to map R loops formation by using the S9.6 monoclonal antibody [94,95], but the specificity of the S9.6 antibody for R loops is questioned recently. Another approach, R loop chromatin immunoprecipitation (R-ChIP), is performed to detect R loops distribution by taking advantage of inactive exonuclease RNase H1 for specific DNA-RNA hybrids [96]. Wang et al. developed an R loop CUT&Tag with the N-terminal hybrid-binding domain (HBD) of RNase H1, specifically recognizing DNA-RNA hybrids. R loop CUT&Tag was demonstrated to be sensitive, convenient and reproducible for genuine R loop landscapes with high resolution [97]. Another group performed G4 and R loop CUT&Tag, illustrating the genome-wide co-formation of G4s, single-stranded DNA and R loops at enhancers and promoters in mouse embryonic stem cells [92].

## Conclusion and perspective

Epigenetics has undergone a radical change due to large deep sequencing advances and dramatic reductions in the cost of short-read sequencing. CUT&Tag is a relatively novel chromatin profiling method that has rapidly gained popularity in the field of epigenetics since 2019. It is a modification of the ChIP technique, which is commonly used to study the interaction of proteins with DNA. Compared to ChIP-seq, CUT&Tag has multiple advantages: (a) CUT&Tag only requires the low starting material of 100 cells, while ChIP-seq is commonly performed with more than 1 million cells. (b) CUT&Tag profiles for the genome landscape of TFs and chromatin modification with high signal-to-noise ratio and low background. (c) CUT&Tag is established by Tn5 tagmentation without crosslinking and sonication. Therefore, epitope masking is not an issue for CUT&Tag. (d) The low sequencing depths and base-pair resolution are the features of CUT&Tag. (e) Since Tn5 tightly binds to the targeted DNA fragment, making the DNA fragments stay in chromatin in solution, CUT&Tag is relatively suitable for single-cell chromatin profiling. (f) Spike-in DNA can be used to quantify the enrichment alteration of protein in CUT&Tag. However, it has some limitations that should be taken into consideration. The CUT&Tag method relies on antibodies to target specific chromatin regions for cleavage and labelling. Thus, the specificity of the antibody may vary depending on the experiment and can lead to potential false negatives or false positives. The quality of the samples determines the result of the experiment, which currently requires fresh or freshly frozen samples, which can be a challenge for researchers working with archival or rare samples. Another big issue is the biased Tn5 enzyme, a study reported that Tn5 transposase is prone to target open chromatin. To prevent pA-Tn5 from targeting accessible sites, 300 mM NaCl is used in incubation, tagmentation and washes of CUT&Tag, where high salt competes for Tn5 binding to DNA. This is unfavourable for the detection of TFs because of TF instability in this situation.

**Figure 3.**
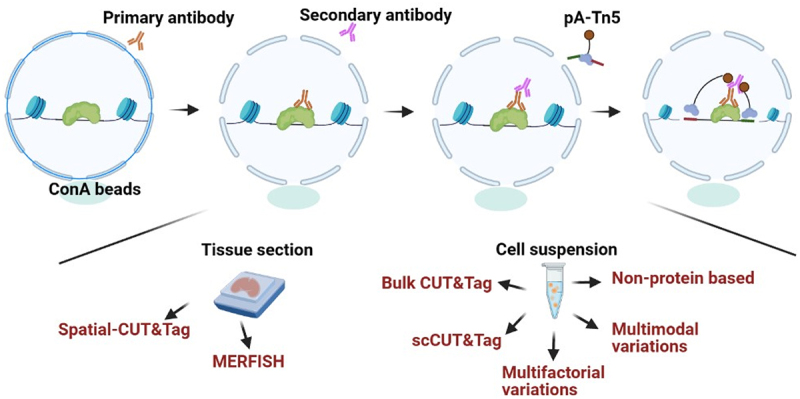
The principle of CUT&Tag and CUT&Tag variants. In the CUT&Tag experiment, the primary antibody recognizes the target protein and then mediates the binding of the secondary antibody and the protein A/G-Tn5 fusion protein. Tn5 can precisely target and cleave the DNA sequence near the target protein in the presence of Mg^2+^. Simultaneously, the sequencing adapters will be inserted into both ends of DNA fragments by Tn5. For tissue section, spatial-CUT&Tag and MERFISH can be conducted. For cell suspension, CUT&Tag and CUT&Tag variants can be performed.

CUT&Tag has been widely adapted to map chromatin modifications and TFs in different species, illustrating the association of these chromatin epitopes with various physiological and pathological processes. Scalable sc-CUT&Tag can be combined with distinct platforms, including droplet, nanowell and split-pool platforms, to distinguish cellular identity and epigenetic features in a single cell. Compared with split-pool sc-CUT&Tag, droplet and nanowell-based sc-CUT&Tag are easier to automate and commercialize, but more expensive instruments are required. Although epigenome analysis is limited to a maximum of two copies of chromatin characteristics per diploid cell, the CUT&Tag derivative has been developed to increase cell type discriminatory information of each cell by using T7 promoter-mediated linear amplification. Therefore, the advantage of Tip-seq is increasing the number of fragments per cell to identify more epigenetic features, however, the experiment procedure become more tedious and complicated. Integration of multiomics data is promising for elucidating biological mechanisms from multiple levels and dimensions. CUT&Tag is not only simply combined with other approaches but also developed as a strategy for joint profiling of the epigenome, transcriptome or proteome on the same sample, which facilitate to distinguish cellular identity and get more information from rare sample. Multifactorial CUT&Tag approaches simultaneously allow profiling for multiple histone modifications and chromatin binding proteins with distinct strategies such as computational analysis, the addition of barcode adaptors in primary antibody or pA-Tn5, and the use of nanobodies. These multifactorial CUT&Tag approaches show different strengths and weaknesses. For example, CUT&Tag 2 in 1 is only distinguish specific histone modifications and TFs with the distinct breadth of binding sites. Multi-CUT&Tag and MulTI-Tag can observe more histone modifications and TFs, but the adaptors of Tn5 should be reconstructed. NanoCUT&Tag requires nano antibodies which are specially purchased from specific companies. Although some strategies for spatial CUT&Tag have been designed, the commercial availability of spatial CUT&Tag may need continuous improvement. Indeed, multifactorial and multimodal CUT&Tag will offer new perspectives for spatial CUT&Tag. The analytical approaches for CUT&Tag are derived from ChIP-seq, CUT&RUN, scRNA-seq and scATAC-seq [98,99]. However, more novel analytical tools that are suitable for multifactorial, multimodal CUT&Tag and spatial CUT&Tag will be developed. Nonprotein features of chromatin are also investigated by CUT&Tag, including DNA G-quadruplexes, RNA loops and RNA methylation [100]. We have summarized CUT&Tag and CUT&Tag variants in Table 1 (Figure 3), as well as the combinations of CUT&Tag with other omics in Table 2 (Figure 3), and we do believe CUT&Tag and derivatives will be adapted to wider application scenarios. Furthermore, with the development of automation technology, CUT&Tag can also be automated, reducing processing time and human error [12]. The future outlook for the CUT&Tag method is very promising, as it has become an important tool in scientific and clinical research.

**Table 1.** CUT&Tag and CUT&Tag variants.

Technique	Antibody	Feature	Simultaneous profiling multiple proteins	Minimal cell number	Single cell detection	Spatial single-cell detection	Ref
CUT&Tag	Primary, secondary	Tn5-Protein A fusion	NO	1–60	YES	YES	[10]
scCUT&Tag	Primary, secondary	Single-cell resolution	NO	1	YES	YES	[10], [54], [61], [62]
TIP seq	Primary, secondary	T7 transcription based scCUT &Tag	NO	1	YES	Likely	
CUT&Tag 2 in 1	Primary, secondary	Bulk and single-cell deconvolution CUT&Tag	YES	1	YES	Likely	[70]
multi-CUT&Tag	Primary, secondary	Multifactorial bulk and single-cell CUT&Tag	YES	1	YES	Likely	[71]
MulTI-Tag	Primary, secondary	Multifactorial bulk and single-cell CUT&Tag	YES	1	YES	Likely	[72]
NanoCUT&Tag	Primary, nanobody	Multifactorial single-chain antibodies fused to Tn5	YES	1	YES	Likely	[73], [74]

**Table 2.** The combinations of CUT&Tag with other omics.

Technique	Antibody	Feature	Simultaneous profiling multiple proteins	Minimal cell number	Single cell detection	Spatial single-cell detection	Ref
CoTECH	Primary, secondary	Joint scCUT &Tag and scRNA-seq	NO	1	YES	Likely	[65]
Paired-Tag	Primary, secondary	Joint scCUT &Tag and scRNA-seq	NO	1	YES	Likely	[67]
scCUT&Tag-pro	Primary, secondary	Joint scCUT &Tag and abundance of surface proteins	NO	1	YES	Likely	[68]
CUT&Tag-BS	Primary, secondary	CUT&Tag with bisulphite sequencing	NO	4000	NO	NO	[69]
Spatial-CUT&Tag	Primary, secondary	NGS based Spatial-CUT&Tag	NO	1	YES	YES	[75]
epigenomic MERFISH	Primary, secondary	Multiplexed imaging based Spatial-CUT&Tag	NO	1	YES	YES	[76]

## Data Availability

Data sharing is not applicable to this article as no new data were created or analysed in this study.
